# *The Fibroblast Growth Factor 9 (Fgf9)* Participates in Palatogenesis by Promoting Palatal Growth and Elevation

**DOI:** 10.3389/fphys.2021.653040

**Published:** 2021-04-20

**Authors:** Ruomei Li, Yidan Sun, Zhengxi Chen, Mengting Zheng, Yuhua Shan, Xiyu Ying, Mengjia Weng, Zhenqi Chen

**Affiliations:** ^1^Department of Orthodontics, Shanghai Key Laboratory of Stomatology, Shanghai Ninth People’s Hospital, Shanghai Jiao Tong University School of Medicine, Shanghai Jiao Tong University, Shanghai, China; ^2^Resident, Department of General Dentistry, Henry M. Goldman School of Dental Medicine, Boston University, Boston, MA, United States

**Keywords:** secondary palate development, fibroblast growth factor 9, hyaluronic acid, mandibular growth, tongue movement

## Abstract

Cleft palate, a common global congenital malformation, occurs due to disturbances in palatal growth, elevation, contact, and fusion during palatogenesis. The *Fibroblast growth factor 9* (*FGF9*) mutation has been discovered in humans with cleft lip and palate. *Fgf9* is expressed in both the epithelium and mesenchyme, with temporospatial diversity during palatogenesis. However, the specific role of *Fgf9* in palatogenesis has not been extensively discussed. Herein, we used *Ddx4-Cre* mice to generate an *Fgf9^–/–^* mouse model (with an *Fgf9* exon 2 deletion) that exhibited a craniofacial syndrome involving a cleft palate and deficient mandibular size with 100% penetrance. A smaller palatal shelf size, delayed palatal elevation, and contact failure were investigated to be the intrinsic causes for cleft palate. Hyaluronic acid accumulation in the extracellular matrix (ECM) sharply decreased, while the cell density correspondingly increased in *Fgf9^–/–^* mice. Additionally, significant decreases in cell proliferation were discovered in not only the palatal epithelium and mesenchyme but also among cells in Meckel’s cartilage and around the mandibular bone in *Fgf9^–/–^* mice. Serial sections of embryonic heads dissected at embryonic day 14.5 (E14.5) were subjected to craniofacial morphometric measurement. This highlighted the reduced oral volume owing to abnormal tongue size and descent, and insufficient mandibular size, which disturbed palatal elevation in *Fgf9^–/–^* mice. These results indicate that *Fgf9* facilitates palatal growth and timely elevation by regulating cell proliferation and hyaluronic acid accumulation. Moreover, *Fgf9* ensures that the palatal elevation process has adequate space by influencing tongue descent, tongue morphology, and mandibular growth.

## Introduction

Cleft lip and/or palate is a common congenital birth defect affecting approximately 1 in 700 newborns (Dixon et al., 2011). The prevalence of cleft palate is 0.13–2.53‰ worldwide (Burg et al., 2016). Mice with cleft palate share similar developmental and genetic features with humans with cleft palate (Mittwoch, 2008). Secondary palate formation in both species is generally divided into three periods, comprising palatal growth and enlargement before embryonic day 13.5 (E13.5), elevation and contact before E14.5, and fusion before E15.5 (Li et al., 2019). Thus, effort has been made to explore the possible genetic factors responsible for cleft palate in mice, which represent the optimum model for identifying the underlying mechanism in humans (Juriloff and Harris, 2008; Burg et al., 2016; Li et al., 2019).

The 22 members of the fibroblast growth factor (FGF) family participate in multiple aspects of palate development. Mutations in these factors are involved in 3–5% of cases of non-syndromic cleft lip and palate in humans (Riley et al., 2007; Wu et al., 2015; Jin et al., 2018; Weng et al., 2018). The *fibroblast growth factor 9* (*FGF9*) T > c mutation has been reported in a non-syndromic bilateral cleft lip and palate case (Riley and Murray, 2007). Moreover, cleft palate has been observed in *Fgf9*^–/–^ mice with 40% penetrance, but the underlying mechanism has not been elaborated (Colvin et al., 2001).

*Fgf9* is located on chromosome 14qC3, and it was initially recognized as a trophic factor for primary rat glial cells (Naruo et al., 1993). Moreover, it is also known as heparin-binding growth factor 9 and it has an affinity for fibroblast growth factor receptors 2 and 3 (FGFR2 and FGFR3) and heparin (Hecht et al., 1995; Harada et al., 2009). It was initially detected in the maternal uterine epithelium at E7.5 (Colvin et al., 1999). From E9.5 to E12.5, it is expressed in the ectoderm of the craniofacial region, with spatiotemporal variation (Colvin et al., 1999). It is then detected in the palatal epithelium at E13.5, and in both the epithelium and mesenchyme at E14.5 during palatal elevation (Iwata et al., 2012). Additionally, *Fgf9* is involved in genetic cross talk with the Sox, Fgf, and Has families, all of which have unique and important functions in palatal growth and elevation (Lee and Saint–Jeannet, 2011; Loke et al., 2014; Chang et al., 2018; Wolk et al., 2020; Yonemitsu et al., 2020; Yue et al., 2020). Thus, it is rational to propose that *Fgf9* plays an essential role during palatogenesis and possibly mediates palatal growth and elevation.

To verify our hypothesis and explore *Fgf9*’s possible functions, we generate an *Fgf9* knockout murine model with 100% penetrance of cleft palate. Further, we provide evidence that *Fgf9* mediates palatal growth and elevation basing on the examinations of histologic measurements, hyaluronic acid accumulation, cell proliferation, cell apoptosis analysis, etc.

## Materials and Methods

### Generation of Transgenic Mice

**FIGURE 1:**
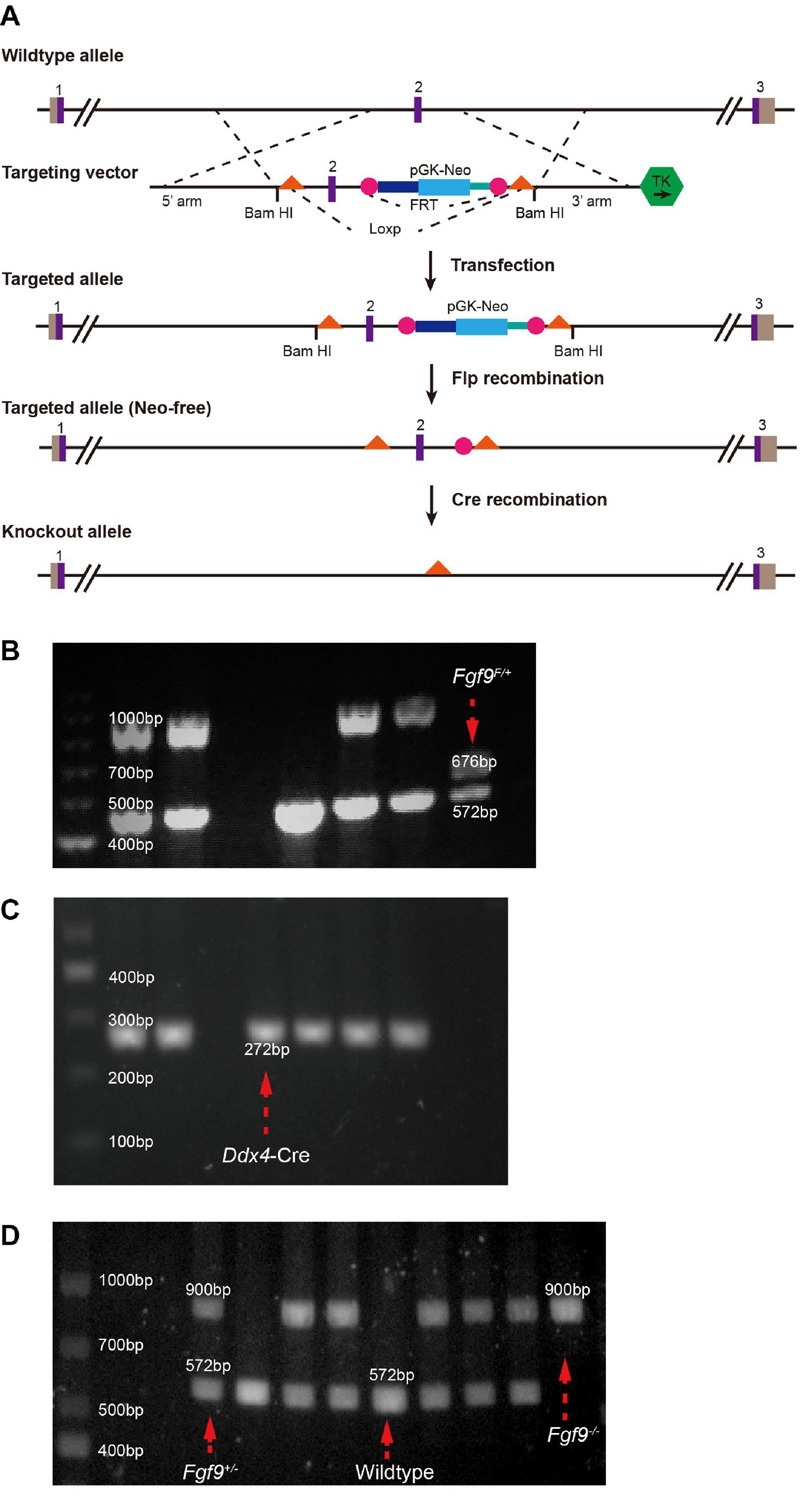
Generation of *Fgf9* knockout allele. **(A)** Generations of *Fgf9* allele lacking exon 2. Purple boxes, exons. Gray boxes, introns. Orange triangles, loxP sites. pGK-NEO, pGK-NEO cassette for positive selection. TK, thymidine kinase cassette included for negative selection. PCR-based genotyping of **(B)** *Fgf9*^*F/+*^ (F indicates a floxed allele), **(C)** *Ddx4–Cre*, **(D)** *Fgf9^+/–^*, *Wildtype*, and *Fgf9^–/–^* mice.

The strategy of generating an *Fgf9* knockout allele is illustrated in Figure 1A. The recombinant vector of exon 2 (Fgf9–201, ENSMUST00000022545.13) of mouse *Fgf9* (MGI: 104723) was constructed and transfected into SM–1 embryonic stem (ES) cells by electroporation. The positive ES cells were screened by G418 (350 Ag/mL) and ganciclovir (2 mmol/L) and then identified by Southern blotting. ES cells were microinjected into 102 blastocysts of C57BL/6J mice and then transferred into the uterus of pseudopregnant mice to obtain chimeric mice. Seven chimeric mice were identified and mated with *Wildtype* C57BL/6J mice. Four flox-NEO alleles (*Fgf9*^*FloxNeo*^) were selective from the F1 agouti offspring by PCR. Neo-free mice (*Fgf9^*flox/+*^; Flp^+^*) were generated by mating *Fgf9*^*FloxNeo*^ with Flp mice. Finally, *Fgf9*^*flox/+*^ (*Fgf9^F/+^*) were generated by crossing *Fgf9^*flox/+*^; Flp^+^* with *Wildtype*. *Fgf9^F/+^* mice were sequenced to confirm correct targeted sites (forward primer CACTGGGCTCTAACTCTTC and reverse primer GACAATAATTTCCACCTCC) (Figure 1). Mice are housed in accredited animal facilities in specific pathogen-free conditions of Shanghai Ninth People’s Hospital (Shanghai, China).

*Ddx4-Cre* mice purchased from Shanghai Model Organisms Center, Inc, Shanghai, China, were crossed with *Fgf9^F/+^* to obtain *Fgf9^*F/+*^; DDX4-Cre* mice (Gallardo et al., 2007) (Figure 1C). *Fgf9^*F/+*^; DDX4-Cre* mice were crossed with Wildtype mice to generate *Fgf9^+/–^* mice. Moreover, male *Fgf9^+/–^* was crossed with female *Fgf9^+/–^* to generate *Fgf9^–/–^*, *Fgf9^+/–^*, and *Wildtype* embryos. Mice were genotyped by PCR (primer 1 ATTTGCTATGCACGGACAC, primer 2 CACTGGGCTCTAACTCTTC, and primer 3 GACAATAATTTCCACCTCC). Briefly, a 900-bp band and a 572-bp band represent *Fgf9-null* and *Wildtype* alleles, respectively (Figure 1D). The morning that the vaginal plug was identified was designated as embryonic day 0.5 (E0.5). All experiments were repeated on at least three littermates per genotype. The representative tissue of each genotype was chosen at random. Timed pregnant mice were euthanized using CO_2_, followed by thoracotomy to ensure death. The uterus with embryos was removed, and embryos were retrieved under a microscope. The tissues were further dissected and processed for analysis, as described below. The experience was undergone according to the protocols of the Animals’ Committee of Shanghai Ninth People’s Hospital (Shanghai, China).

### Micro-CT Analysis and Skeletal Staining of Mouse Skulls

Skulls were isolated and fixed in 70% ethanol and scanned using SkyScan 1076 (Belgium) with a spatial resolution of 24 μm. Mice obtained at E18.5 were sacrificed, skinned, eviscerated, and fixed in 95% ethanol overnight and acetone overnight at room temperature before processing and staining with alcian blue and alizarin red, according to the protocols described by Ovchinnikov (2009).

### Hematoxylin and Eosin (HE) Staining and Masson’s Trichrome (MASSON) Staining

Embryonic heads harvested from timed pregnant female mice were fixed in 4% paraformaldehyde (PFA) at 4°C. Samples were cut to a thickness of 5 μm. Moreover, sections through palatal shelves were selected from the anterior, middle, and posterior regions, with the middle region consisting of sections through the maxillary first molar tooth buds. HE staining was performed using standard procedure. For MASSON staining, sections were stained with Masson’s Trichrome Stain Kit (Solarbio, China). Briefly, the sections were treated sequentially with Bouin’s solution for 15 min, Weigert’s working hematoxylin for 10 min, Biebrich scarlet-acid fuchsin for 5 min, phosphotungstic/phosphomolybdic acid for 10 min, and aniline blue for 5 min.

### *In situ* Hybridization

Embryos were collected at desired developmental stages and fixed in 4% paraformaldehyde overnight at 4°C. For section *in situ* hybridization, embryos were dehydrated through graded alcohols, embedded in paraffin, and sectioned at 7 μm. The sections through palatal shelves were selected from the anterior, middle, and posterior regions, with the middle region consisting of sections through the maxillary first molar tooth buds. The *Fgf9* probe sequence was a generous gift from Dr. David M Ornitz (Colvin et al., 1999). Enhanced Sensitive ISH Detection Kit II (MK1032, Boster, China) was applied for staining. Sections were treated with proteinase K (in prewarmed 50 mM Tris for 10–20 min at 37°C) for antigen retrieval. Following the pre-hybridization step, the sections were incubated in a hybridization solution (2 μg/ml) at 37°C overnight. The slides were treated with a blocking buffer at 37°C for 30 min. Furthermore, biotinylated digoxin was applied at 37°C for 2 h.

### Cell Proliferation Assay, Cell Apoptosis Assay, and Hyaluronic Acid-Binding Protein (HABP) Staining

Proliferation assays were performed by intraperitoneal injection of bromodeoxyuridine (BrdU) labeling reagent (100 μg/g body weight) into pregnant mice, which were sacrificed after 2 h, and embryos recovered and processed. Tissue sections from the comparable anterior, middle, and posterior palatal regions were stained with an antibody to BrdU (1:100, Abcam, United Kingdom). Sections were counterstained with DAPI solution for 7 min to visualize nuclei and were then rinsed in PBS. Two non-serial sections were counted for each region from three *Wildtype*, three *Fgf9^+/–^*, and three *Fgf9^–/–^* littermates at E13.5 and E14.5. Moreover, the mean values were recorded using ImageJ (MD, United States). Palatal shelves were divided into mesenchyme and epithelium for counting. BrdU-labeled cells were counted and calculated as the percentage of labeled cells among total nuclear-stained cells. *P* < 0.05 was considered as statistically significant (Student’s *t*-test). The terminal deoxynucleotidyl transferase dUTP nick end labeling (TUNEL) assay was performed using the Cell Apoptosis Detection Kit IV (CY3) (MK1016, Hubei, China) following the manufacturer’s instructions. Briefly, tissues were fixed in 4% paraformaldehyde and then dehydrated through an increasing graded ethanol series and processed for sectioning. Following rehydration steps, the sections were treated with Proteinase K (in 10 mM Tris–HCl, pH 8.0 for 10–15 min 37°C). The samples were incubated with TdT and DIG-d-UTP at 37°C for 2 h and a blocking buffer for 30 min at room temperature. Biotinylated anti-digoxin antibody (1:100) was applied for 30 min at 37°C. Sections were counterstained with DAPI solution for 7 min to visualize nuclei and were then rinsed in PBS. To evaluate the expression pattern of hyaluronic acid in the palate shelves at E13.5 and E14.5, the sections were stained with biotin-labeled HABP (1:200, 385911-50UG, Millipore, United States) and detected using Texas Red X-conjugated streptavidin (1:200, S6370, Thermo, United States). Images were taken using an Olympus fluorescence microscope.

### Craniofacial Morphometric Measurements

The selected serial sections of embryonic heads from three different litters at E14.5 were applied for craniofacial morphometric measurements. The oral volume, tongue size, tongue height, tongue width, Intra-Meckel’s width, and oral height are evaluated.

### Statistical Analysis

All quantitative data were presented as the mean ± SD as indicated by at least three independent experiments. IBM SPSS Statistics 25.0 (United States) was applied for analysis. *P* < 0.05 was considered as statistically significant (Student’s *t*-test).

## Results

### Fgf9^–/–^ Embryos Exhibited Cleft Secondary Palate and Craniofacial Deformities

**FIGURE 2:**
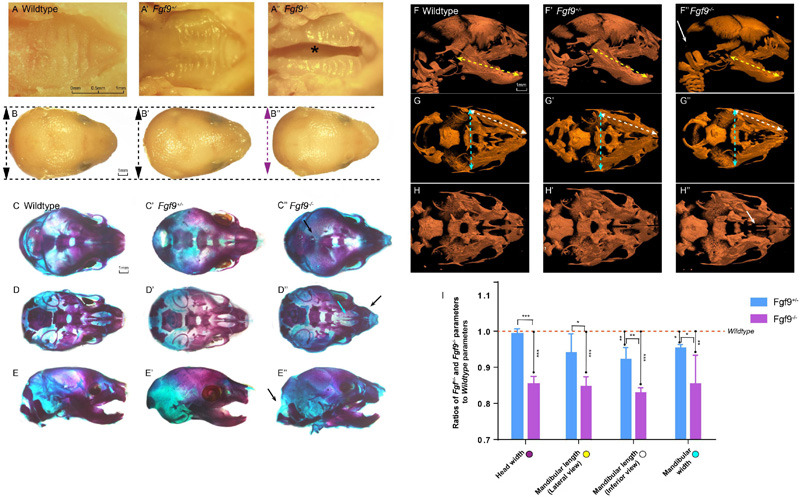
Craniofacial phenotypes of *Wildtype*, *Fgf9^+/–^*, and *Fgf9^–/–^* mice. Inferior views of **(A)** *Wildtype*, **(A’)** *Fgf9^+/–^*, and **(A”)** *Fgf9^–/–^* E18.5 embryos. “*” in **(A”)** indicates cleft secondary palate. Top views of **(B)** *Wildtype*, **(B’)** *Fgf9^+/–^*, and **(B”)** *Fgf9^–/–^* heads. Purple double-headed arrow in **(B”)** indicates narrow head width. Black double-headed arrows in **(B,B’)** indicate normal head widths. Top views of the skeletal staining of **(C)** *Wildtype*, **(C’)** *Fgf9^+/–^*, and **(C”)** *Fgf9^–/–^* heads. Black arrow in **(C”)** indicates the premature partial ossification. Inferior views of the skeletal staining of **(D)** *Wildtype*, **(D’)** *Fgf9^+/–^*, and **(D”)** *Fgf9^–/–^* heads. Blue arrow in **(D”)** indicates cleft secondary palate. Black arrow in **(D”)** indicates the abnormal circular tip of the premaxilla. Lateral views of the skeletal staining of **(E)** *Wildtype*, **(E’)** *Fgf9^+/–^*, and **(E”)** *Fgf9^–/–^* heads. Black arrow in **(E”)** indicates occipital deficiency. Lateral views of **(F)** *Wildtype*, **(F’)** *Fgf9^+/–^*, and **(F”)** *Fgf9^–/–^* embryos showing mandibular lengths (yellow double-headed arrows) on micro-CT scans. White arrows in **(F”)** indicate the occipital deficiency. Inferior views of **(G)** *Wildtype*, **(G’)** *Fgf9^+/–^*, and **(G”)** *Fgf9^–/–^* embryos showing mandibular widths (blue double-headed arrows) and mandibular lengths (white double-headed arrows) on micro-CT scans. Inferior views of **(H)** *Wildtype*, **(H’)** *Fgf9^+/–^*, and **(H”)** *Fgf9^–/–^* embryos. White arrow in **(H”)** indicates the cleft secondary palate. **(I)** Ratios of *Fgf9^+/–^* and *Fgf9^–/–^* parameters to *Wildtype* parameters. *P < 0.1, **P < 0.05, ***P < 0.001.

By dissecting pregnant *Fgf9^+/–^* mice that had mated with male *Fgf9^+/–^* mice, we harvested *Fgf9^–/–^* embryos which died shortly after birth with a significant cleft secondary palate (black star, 100% phenotype penetrance, *n* = 12, Figures 2A–A”). The inferior view of the *Fgf9^–/–^* palate showed a narrower palatal width, with palatal rugae (Figures 2A–A”). The top view of the *Fgf9^–/–^* mice showed a smaller head width (purple double-headed arrow) compared to their *Wildtype* and *Fgf9^+/–^* littermates (black double-headed arrow, Figures 2B–B”).

The skeletal staining and micro-computed tomography (CT) further showed the separation of the palatine and maxillary shelves, enabling a direct view of the vomer and presphenoid (Figures 2D”,H”). Premature parietal ossification can be seen in Figure 2C”. The abnormal circular tip of the premaxilla (black arrow) can be viewed distinctly based on the skeletal staining (Figure 2D”). Moreover, occipital deficiency was observed (Figures 2E”,F”). Several parameters, involving mandible length from lateral views (yellow double-headed arrows), mandibular length from inferior views (white double-headed arrows), and mandibular width from inferior views (green double-headed arrows), were analyzed based on micro-CT reconstruction (Figures 2F–H”). The ratios of the *Fgf9^+/–^* or *Fgf9^–/–^* parameters to the *Wildtype* parameters are displayed in Figure 2I. The head width (85.3% ± 1.2%), mandibular length (lateral view, 83.9% ± 2.7%; inferior view, 83.6% ± 1.8%), and mandibular width (85.4% ± 7.1%) were about 15% lower in the *Fgf9^–/–^* mice compared to the *Wildtype* mice. These results revealed a successfully established *Fgf9* knockout mouse model exhibiting cleft secondary palate, smaller mandible size, occipital deficiency, premature parietal ossification, and malformed premaxilla. The 100% penetrance of cleft secondary palate provided an excellent foundation for exploring the function of *Fgf9* during palatogenesis.

### *Fgf9^–/–^* Embryos Displayed Defective Palatal Growth and Delayed Palatal Elevation

**FIGURE 3:**
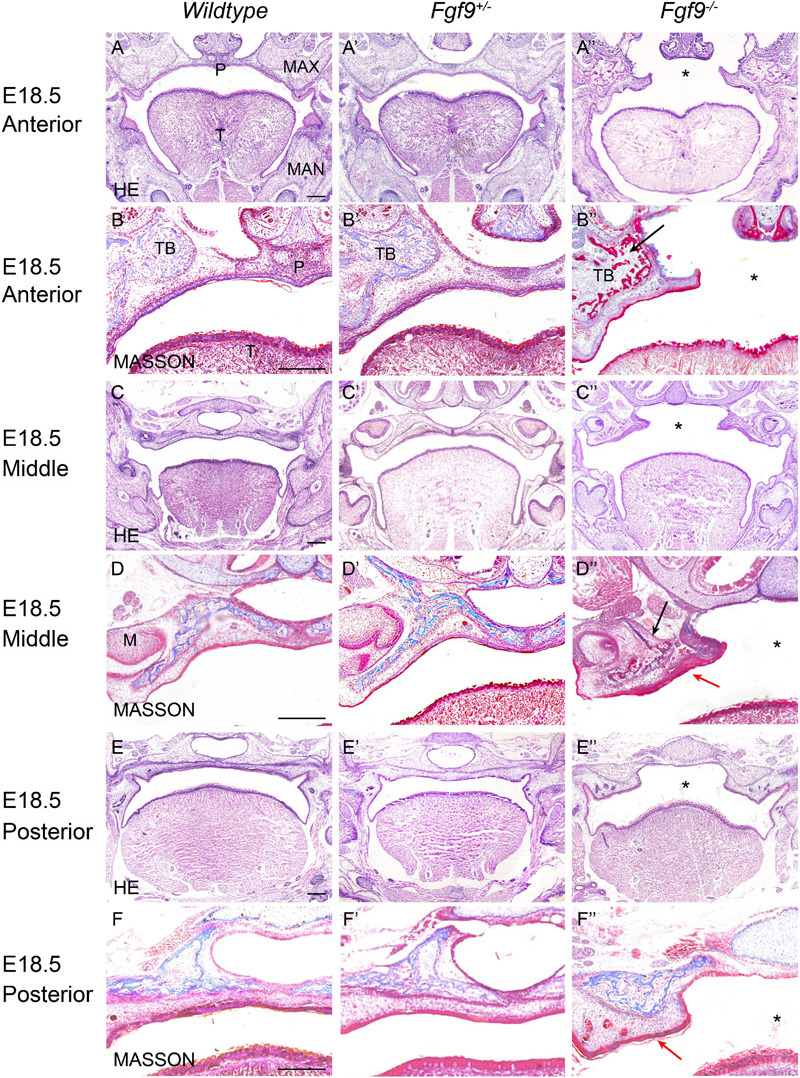
Aberrant morphology and fusion failure in *Fgf9^–/–^* embryos at E18.5. The HE staining of **(A–A”)** anterior, **(C–C”)** middle, and **(E–E”)** posterior palate in Wildtype, *Fgf9^+/–^* and *Fgf9^–/–^* embryos The magnified view showing MASSON staining of **(B–B”)** anterior, **(D–D”)** middle, and **(F–F”)** posterior palate in *Wildtype*, *Fgf9^+/–^*, and *Fgf9^–/–^* embryos. Black arrows indicate the premature trabecular bone development. Red arrows indicate the thickened cuticular layer. *, cleft secondary palate. P, palate. MAX, maxilla. MAN, mandible. T, tongue. TB, trabecular bone. M, molar. Scale bars = 200 μm.

**FIGURE 4:**
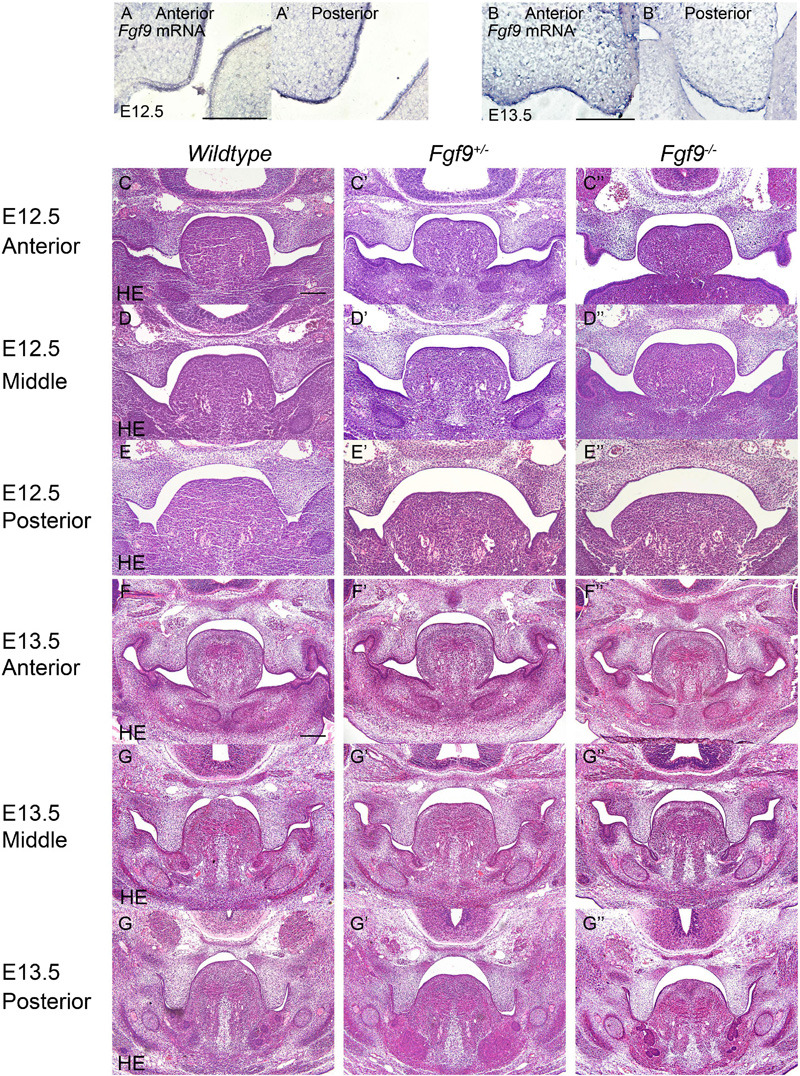
*Fgf9* expression and phenotypes of *Wildtype*, *Fgf9^+/–^*, and *Fgf9^–/–^* embryos at E12.5 and E13.5. Location of *Fgf9* mRNA at E12.5 in **(A)** anterior and **(A’)** posterior palatal shelves, and at E13.5 in **(B)** anterior and **(B’)** posterior palatal shelves. *Fgf9^–/–^* embryos exhibited no significant abnormalities at **(C–E”)** E12.5 and **(D–G”)** E13.5. Scale bars = 200 μm.

**FIGURE 5:**
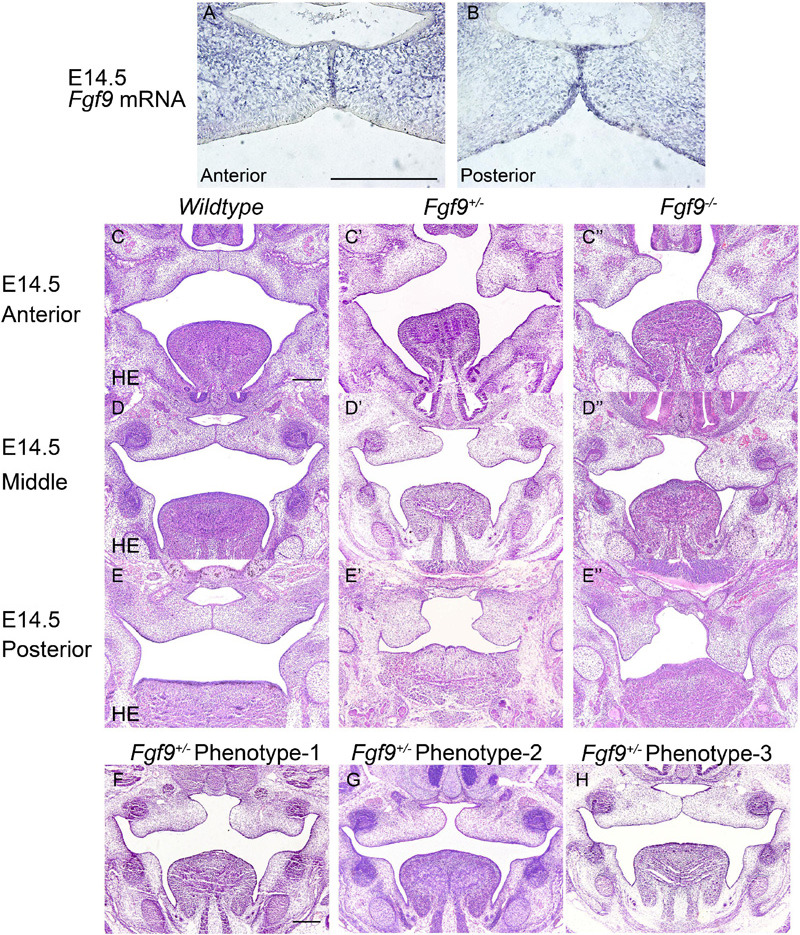
*Fgf9* expression and phenotypes of *Wildtype*, *Fgf9^+/–^*, and *Fgf9^–/–^* embryos at E14.5. Location of **(A)** *Fgf9* mRNA and **(B)** FGF9 protein in anterior and posterior regions at E14.5. HE staining of of **(C–C”)** anterior, **(D–D”)** middle, and **(E–E”)** posterior palate in *Wildtype*, *Fgf9^+/–^*, and *Fgf9^–/–^* embryos at E14.5. **(F–H)** HE staining of various types of delayed palatal elevation in *Fgf9^–/–^* embryos. Scale bars = 200 μm.

**FIGURE 6:**
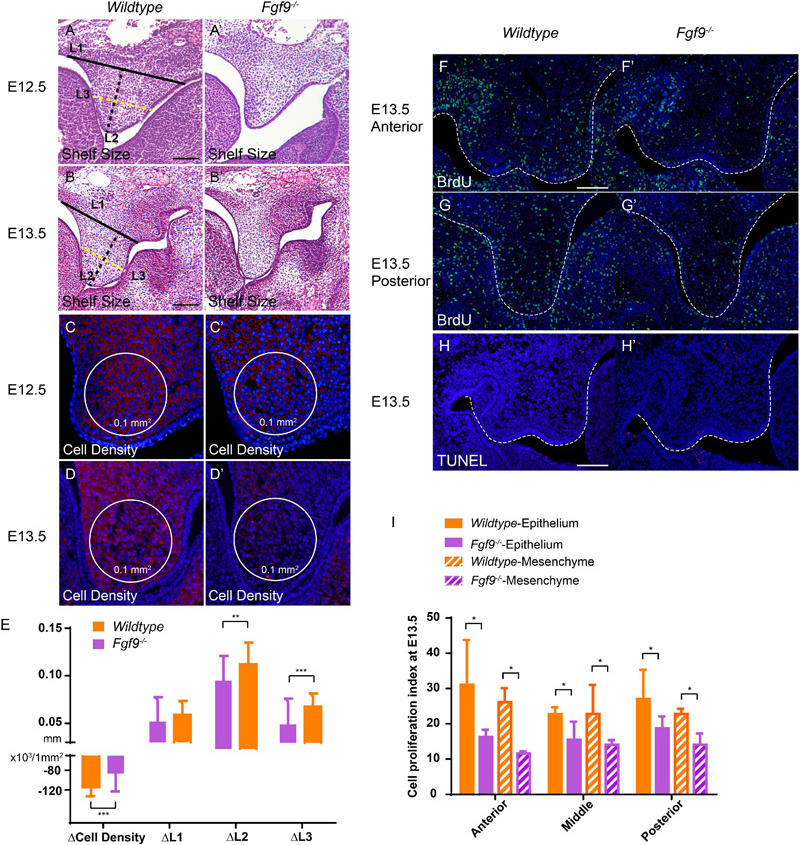
Fgf9^–/–^ palatal shelves exhibit attenuated size increase and cell density decrease from E12.5 to E13.5. Selected serial sections of the anterior (*n* = 7), middle (*n* = 8), and posterior (*n* = 5) of the embryonic heads from three litters were measured. HE staining shows smaller palatal shelves in the Fgf9^–/–^ embryos than the *Wildtype* littermates at **(A,A’)** E12.5 and **(B,B’)** E13.5. L1, L2, and L3 are shorter in the Fgf9^–/–^ embryos. Cell density was higher in the Fgf9^–/–^ embryos than the *Wildtype* littermates at **(C,C’)** E12.5 and **(D,D’)** E13.5. L1, L2, and L3 were shorter in the Fgf9^–/–^ embryos. The cell density and palatal shelf size of Fgf9^–/–^ embryos exhibited attenuated reduction and less growth, respectively **(E)**. Coronal sections with BrdU immunofluorescence through **(F,F’)** anterior and **(G,G’)** posterior regions of *Wildtype* and Fgf9^–/–^ embryos at E13.5. Coronal sections with TUNEL assays of **(H)** *Wildtype* and **(H’)** Fgf9^–/–^ embryos at E13.5. **(I)** Quantification of cell proliferation index through anterior, middle, and posterior regions in the epithelium and mesenchyme of the *Wildtype* and Fgf9^–/–^ embryos at E13.5. Full lines, black dotted lines, and yellow dotted lines indicate the width (L1), length (L2), and 1/2 width (L3) of palatal shelves, respectively. *P < 0.1, **P < 0.05, ***P < 0.001. Scale bars = 100 μm.

The histological analysis showed aberrant morphology, delayed palatal elevation, and fusion failure in the *Fgf9^–/–^* embryos (Figures 3–6).

At E18.5, *Fgf9^–/–^* embryos exhibited elevated and unfused palatal shelves with aberrant morphology (Figures 3A”,C”,E”). In the *Wildtype* embryos, trabecular bone was thick, from the alveolar bone to the middle of the palate. However, the *Fgf9^–/–^* trabecular bone was separated and thin, with premature palatal mineralization, especially in the anterior region (Figures 3B”,D”). Additionally, the cuticular layer of the oral epithelium of the unfused palate was thickened (Figures 3D”,F”).

To identify the causes of the abnormalities, the histological morphologies at crucial time points were evaluated (Figures 4–6). After the emergence of the palatal shelves from the maxillary prominence at E11.5, they grow and enlarge vertically on either side of the tongue (Li et al., 2017). At E12.5 and E13.5, while the vertical outgrowth is ongoing, *Fgf9* mRNA primarily dominated the anterior and posterior epithelia, with a buccal–lingual gradient throughout the palate (Figures 4A–B’). No significant abnormalities were observed except for a relatively smaller size in the *Fgf9^–/–^* embryos (Figures 4C–G”).

In *Wildtype* mice, the palate shelves had elevated to the horizontal position at E14.5, and *Fgf9* mRNA occupied the medial edge epithelium and mesenchyme (where bone formations were being prepared), with dotted expression in the oral epithelium of the palate (Figures 5A,B). In some of the *Fgf9^+/–^* embryos, palatal elevation and contact were delayed, with an asynchronous elevation or without palatal shelf contact (Figures 5F–H). However, intriguingly, at the same time of E14.5, most of the *Fgf9^+/–^* embryos showed normal elevation without palatal shelf contact (Figure 5G). In other *Fgf9^+/–^* embryos, some shelves were in the process of elevation (Figure 5F), and in the remainder of the *Fgf9^+/–^* embryos, successful elevation and contact were observed (Figure 5H). In *Wildtype* palatogenesis, the asynchronous elevation and unfused palatal shelves are considered to be a normal and sequential phenomenon that occurs at around E14.5 (Yu and Ornitz, 2011). In contrast, a great number of the *Fgf9^+/–^* embryos exhibited delayed palatal elevation and shelf contact compared to their *Wildtype* littermates. Regarding the *Fgf9^–/–^* embryos, delayed elevation and aberrant short shelf were observed, probably resulting from impaired palatal shelf remodeling. Moreover, the disharmonious tongue–shelf relationship may act as an obstacle to shelf elevation and/or remodeling to reach the horizontal position (Figures 5C”,D”,E”).

Based on the above evidence, we deduced that palatal shelves lacking *Fgf9* were smaller during the vertical growth, and then palatal elevation, with aberrant shape, was delayed due to poor remodeling and the disharmonious tongue–shelf relationship.

### *Fgf9^–/–^* Palatal Shelves Exhibited Aberrant Cell Proliferation and Increased Cell Density

To determine the immediate cause of the small palatal size in the *Fgf9^–/–^* embryos, we comprehensively assessed the palatal shelf width (L1), length (L2), 1/2 width (L3), and the mesenchymal cell density (Figures 6A–D’). The changes in these parameters in *Wildtype* and *Fgf9^–/–^* mice from E12.5 to E13.5 are shown in Figure 6E. The palatal shelves were smaller (Figures 6A–B’), while the mesenchymal cell density was higher in the *Fgf9^–/–^* embryos (Figures 6C–D’). From E12.5 to E13.5, when the shelves are actively growing and enlarging, the mesenchymal cell density decreased in both groups. Nevertheless, the cell density and the palatal shelf size of *Fgf9^–/–^* mice compared to *Wildtype* mice exhibited attenuated reduction and less growth, respectively (Figure 6E).

To detect the reasons for the above discoveries, we performed bromodeoxyuridine (BrdU) labeling and terminal deoxynucleotidyl transferase dUTP nick end labeling (TUNEL) assays to examine cell proliferation and apoptosis. At E13.5, during the most active cell proliferation phase, many BrdU-labeled cells were located anteroposteriorly in the *Wildtype* palatal shelves. However, *Fgf9^–/–^* palate exhibited decreased BrdU-labeled cells in both the epithelium and mesenchyme (Figures 6F–G’,I). These reductions indicate sharp decreases in cell proliferation, giving rise to a smaller shelf size. The TUNEL assays showed a comparable apoptosis level in the *Fgf9^–/–^* and *Wildtype* mice (Figures 6H,H’). Together, these data show that the increased mesenchymal cell density in the *Fgf9^–/–^* mice owes to neither the impaired cell proliferation nor the abnormal apoptosis.

### Abnormal HA Accumulation and Cell Proliferation Impaired Palatal Growth and Elevation in the *Fgf9^–/–^* Embryos

HA accumulation is believed to expand the ECM and generate osmotic pressure for palatal elevation (Goudy et al., 2010; Yonemitsu et al., 2020). To examine whether the increased cell density and abnormal palatal elevation resulted from insufficient HA content, we detected hyaluronic acid-binding protein (HABP) at E12.5 and E13.5.

**FIGURE 7:**
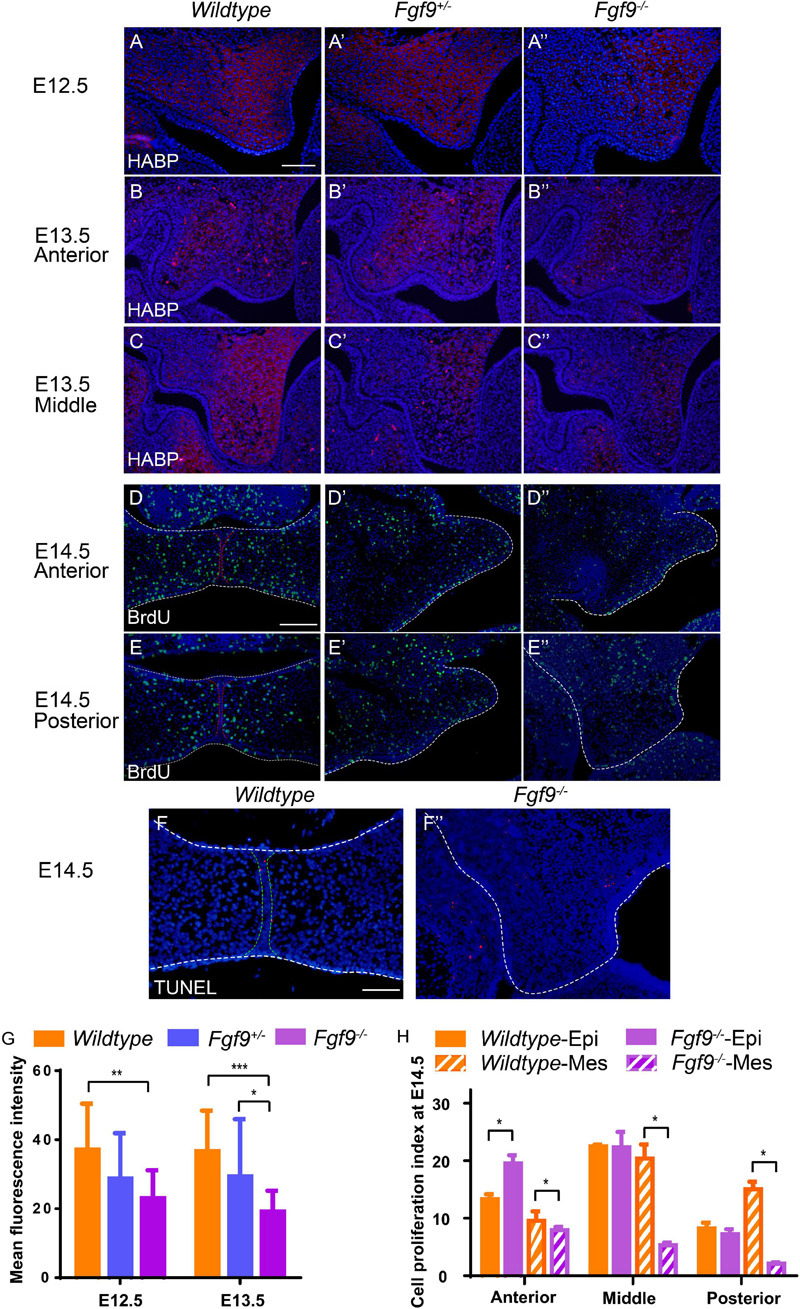
*Fgf9^–/–^* embryos have reduced HA accumulation and cell proliferation. Coronal sections with HABP staining of **(A)** *Wildtype*, **(A’)** *Fgf9^+/–^*, and **(A”)** *Fgf9^–/–^* embryos at E12.5. Coronal sections with HABP staining through **(B–B”)** anterior and **(C–C”)** middle regions of *Wildtype*, *Fgf9^+/–^*, and *Fgf9^–/–^* embryos at E13.5. Coronal sections with BrdU immunofluorescence through **(D–D”)** anterior and **(E–E”)** posterior regions of *Wildtype*, *Fgf9^+/–^*, and *Fgf9^–/–^* embryos at E14.5. Coronal sections with TUNEL assays of **(F)** *Wildtype* and **(F”)** *Fgf9^–/–^* embryos at E14.5. **(G)** Quantification of mean fluorescence intensity in *Wildtype*, *Fgf9^+/–^*, and *Fgf9^–/–^* embryos at E12.5 and E13.5. **(H)** Quantification of cell proliferation index through anterior, middle, and posterior regions in the epithelium and mesenchyme of the *Wildtype* and *Fgf9^–/–^* embryos at E14.5. HABP, hyaluronic acid binding protein. Epi, epithelium. Mes, mesenchyme. *P < 0.1, **P < 0.05, ***P < 0.001. Scale bars = 100 μm.

At E12.5, *Fgf9^–/–^* mice comparing with *Wildtype* and *Fgf9^+/–^* mice exhibited reduced HA accumulation, especially in the anterior region, with a lingual-buccal gradient (Figures 7A–A”). At E13.5, HA accumulation was uniformly decreased in both *Fgf9^+/–^* and *Fgf9^–/–^* mice compared to *Wildtype* mice (Figures 7B–C”). Furthermore, the decrease was steep in the buccal area of the middle and posterior palatal shelves (Figures 7C–C”). The decrease in HA accumulation was more severe at E13.5 in both the *Fgf9^+/–^* and *Fgf9^–/–^* mice, matching the trend in cell density (Figure 7G). Thus, the increased cell density in the *Fgf9^–/–^* mice may be a result of impaired ECM expansion caused by decreased HA accumulation. Subsequently, the increased cell density, together with the impaired cell proliferation, is most likely to be the cause of the aberrant palatal elongation and expansion, leading to a smaller size. Moreover, the aberrant palatal shelves, along with the abnormal osmotic pressure owing to insufficient HA content, impairs palatal elevation. According to the above analysis, *Fgf9* is required for HA accumulation to ensure tissue enlargement and osmotic pressure generation during palatal growth and elevation.

During and after palatal elevation, palatal shelves undergo remodeling and horizontal elongation. At E14.5, the mid-posterior regions of the *Fgf9^–/+^* and *Fgf9^–/–^* palatal shelves were undergoing remodeling (Figures 7E’,E”). The cell proliferation sharply decreased in the *Fgf9^–/–^* palate, especially in the mesenchyme (Figures 7E–E”,H). In the anterior region in the *Fgf9^–/–^* mice, there were higher cell proliferation rates in shelves that had already elevated than in unelevated mid-posterior parts (Figures 7D”,E”). However, there was a lower cell proliferation rate compared to that in the *Fgf9^+/–^* mice, which shared a similar period of horizontal growth after elevation (Figures 7D–D”). The TUNEL assays showed a comparable apoptosis level in the *Fgf9^–/–^* and *Wildtype* mice at E14.5 (Figures 7F,F”).

These results indicate that in addition to the HA accumulation, decreased cell proliferation resulted in abnormal tissue remodeling and insufficient horizontal growth during and after elevation in the *Fgf9^–/–^* palatal shelves.

### Inadequate Oral Volume Owing to Abnormal Tongue Shape and Descent, and Insufficient Mandibular Size in the *Fgf9^–/–^* Mice

**FIGURE 8:**
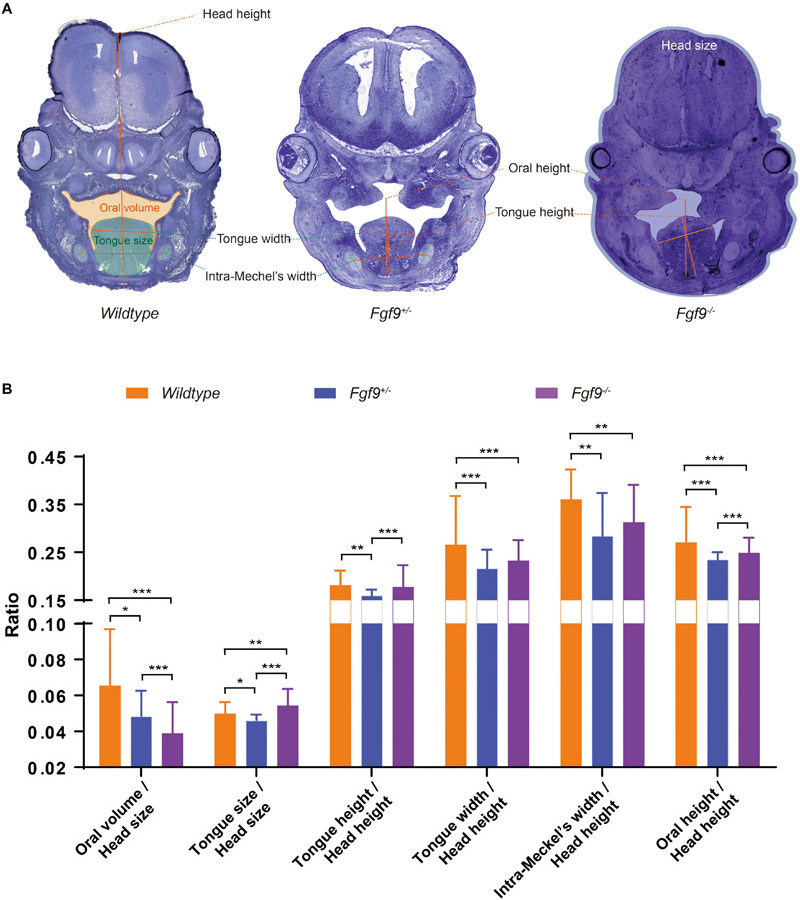
Craniofacial morphometric measurements among *Wildtype*, *Fgf9^+/–^*, and *Fgf9^–/–^* embryos during palatal elevation. **(A)** Oral volume, tongue size, tongue height, tongue width, intra-Meckel’s width (distance between the bilateral centers of Meckel’s cartilages), and oral height based on selected serial sections of embryonic heads from three litters. Orange, green, and blue regions indicate the oral volume, tongue size, and head size, respectively. **(B)** Ratios of craniofacial morphometric parameters. *P < 0.1, **P < 0.05, ***P < 0.001.

To identify factors that may be involved in the aberrant palatal elevation in the *Fgf9^–/–^* embryos, craniofacial morphometric measurements, comprising oral volume, tongue size, tongue height, tongue width, intra-Meckel’s width (distance between the bilateral centers of Meckel’s cartilages), and oral height, were analyzed. The use of selected serial sections in the anterior (*n* = 7), middle (*n* = 8), and posterior (*n* = 5) parts of embryonic heads from three litters for morphometric measurements is illustrated in Figure 8A. Furthermore, the *Fgf9^+/–^* embryos that exhibited asynchronous elevation were investigated to get to the root of the abovementioned disharmonious tongue–shelf relationship.

At E14.5, the palatal shelves had partially “flipped up” or undergone remodeling to reach the horizontal position in the *Fgf9*^+/–^ and *Fgf9^–/–^* embryos (Figure 8A). At this time point, *Fgf9^–/–^* embryos had the smallest oral volume (oral volume/head size, *Fgf9^–/–^* = 0.039 ± 0.008 *vs. Fgf9^+/–^* = 0.052 ± 0.015 *vs. Wildtype* = 0.064 ± 0.023, *P* < 0.05) and the largest tongue size (tongue size/head size, *Fgf9^–/–^* = 0.055 ± 0.003 *vs. Fgf9^+/–^* = 0.046 ± 0.003 *vs. Wildtype* = 0.053 ± 0.005, *P* < 0.05, Figure 8B). The tongue position (tongue height/head height, *Fgf9^–/–^* = 0.174 ± 0.017 *vs. Fgf9^+/–^* = 0.160 ± 0.010, *P* < 0.05) and tongue width (tongue width/head height, *Fgf9^–/–^* = 0.230 ± 0.021 *vs. Fgf9^+/–^* = 0.220 ^ + /−^ 0.023, *P* = 0.152) in the *Fgf9^+/–^* group was lower and shorter, respectively, than in the *Fgf9^–/–^* group. This indicates that timely tongue descent lay the foundation for later palatal elevation (Figure 8B).

The distance between the bilateral centers of Meckel’s cartilages (intra-Meckel’s width/head height) was used to measure the mandibular width, which was shorter in both *Fgf9^+/–^* and *Fgf9^–/–^* embryos compared to the *Wildtype* embryos (*Fgf9^–/–^* = 0.296 ± 0.058 *vs. Fgf9^+/–^* = 0.283 ± 0.099 *vs. Wildtype* = 0.33 ± 0.068, *P* < 0.05, Figure 8B). The oral cavity height (oral height/head height) indicates that the mandibular height was decreased in both *Fgf9^+/–^* and *Fgf9^–/–^* embryos (*Fgf9^–/^* = 0.251 ± 0.020 *vs. Fgf9^+/–^* = 0.230 ± 0.021 *vs. Wildtype* = 0.292 ± 0.037, *P* < 0.05, Figure 8B). These observations might result from the aberrant mandibular development.

These results highlight that delayed tongue movement, insufficient mandibular size, and subsequently inadequate oral volume are impediments to palatal elevation in *Fgf9^–/–^* embryos.

### Reduced Growth Potential in Meckel’s Cartilage and Intramembranous Ossification in the *Fgf9^–/–^* Mice

To investigate the possible reasons for insufficient mandibular growth in the *Fgf9^–/–^* mice, we examined Meckel’s cartilage development and intramembranous ossification.

**FIGURE 9:**
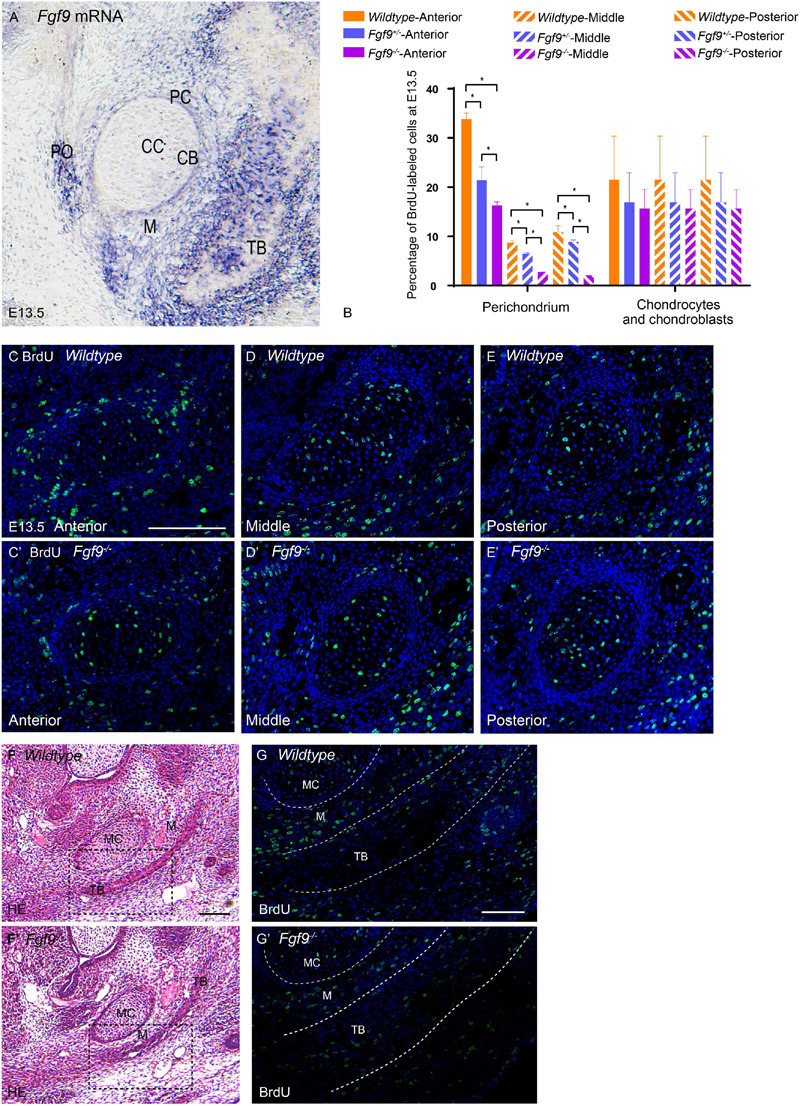
Growth potential of Meckel’s cartilage is impaired in *Fgf9^–/–^* embryos. **(A)** *Fgf9* mRNA is located in the perichondrium, periosteum, trabecular bone, and mesenchyme surrounding bone and cartilage. **(B)** Quantification of BrdU-labeled cells in the perichondrium, chondrocytes, and chondroblasts in different sections of the palate. BrdU immunofluorescence of the anterior part of Meckel’s cartilage in **(C)** *Wildtype* and **(C’)** *Fgf9^–/–^* mice. BrdU immunofluorescence of the anterior part of Meckel’s cartilage in **(D)** *Wildtype* and **(D’)** *Fgf9^–/–^* mice. BrdU immunofluorescence of the anterior part of Meckel’s cartilage in **(E)** *Wildtype* and **(E’)** *Fgf9^–/–^* mice. HE staining of the intramembranous ossification region of **(F)** *Wildtype* and **(F’)** *Fgf9^–/–^* mice. The dotted-box regions in **(F,F’)** were examined with BrdU immunofluorescence, with magnified images in **(G,G’)**, respectively. *P < 0.05. PC, perichondrium. CC, chondrocyte. CB, chondroblast. TB, trabecular bone. PO, periosteum. M, mesenchyme. MC, Meckel’s cartilage. Scale bars = 100 μm.

E13.5, less than a day before palatal elevation in *Wildtype* mice, was explored. At this time point, *Fgf9* mRNA was located in the perichondrium, periosteum, trabecular bone, and mesenchyme surrounding the trabecular bone and cartilage (Figure 9A). This indicates that *Fgf9* may have a role during intramembranous ossification and Meckel’s cartilage development, which is required for appropriate mandibular growth.

The BrdU labeling and TUNEL assays were used to detect cell proliferation and apoptosis, respectively, in Meckel’s cartilage and the surrounding mesenchymal and bone tissue (Figures 9B–E’). In *Wildtype* mice, the ratio of BrdU-labeled cells in the perichondrium was significantly higher in the anterior and posterior parts of the mandible (Figure 9B). This ratio underwent a reduction in the *Fgf9^–/–^* Meckel’s cartilage (Figure 9B).

In comparison to *Wildtype* mice, the *Fgf9^–/–^* trabecular bone at E13.5 was thinner (Figures 9F,F’). The mesenchyme around the *Wildtype* trabecular bone exhibited a high ratio of BrdU-labeled cells. However, the ratio was decreased in the same region of *Fgf9^–/–^* mice (Figures 9G,G’) and in the trabecular bone region of *Fgf9^–/–^* mice (Figures 9G,G’). There was no significant difference in the ratio of apoptotic cells among the three groups (data not shown).

The abovementioned results collectively indicate that the lack of *Fgf9* expression diminished cell proliferation related to the perichondrium and mesenchyme around the trabecular bone, possibly leading to insufficient mandibular growth.

## Discussion

### *Fgf9^–/–^* Mouse Model Is Crucial for Revealing the Genetic Crosstalk in Palatogenesis

Multiple synostoses syndrome and craniosynostosis have been observed in FGF9 mutant mice and humans (Rodriguez–Zabala et al., 2017; Bird et al., 2020; Sentchordi–Montané et al., 2020). However, the syndrome of cleft palate, deficient mandibular size, premature partial ossification, and occipital deficiency in *Fgf9* knockout mice generated in our study has not been reported in mice or humans.

Similar abnormalities of cleft palate and mandibular hypoplasia have been observed to be related to *Sox* family related mutations. *Sox11* mutant mice exhibit cleft palate, cleft lip, and mandibular hypoplasia (Sock et al., 2004; Huang et al., 2016). *Fgf9* was examined as the downstream target of *Sox11*. Moreover, *Fgf9* mutant mice had comparatively milder abnormities compared to *Sox11* mutant mice. In addition, similar phenotypes of premature craniofacial bone mineralization, cleft palate, and deficient mandibular size have been observed in *Sox9* mutant mice (Bi et al., 2001; Lee and Saint–Jeannet, 2011). The palate of *Sox9 + /−* mice exhibited delayed palatal elevation that resembles that in *Fgf9^–/–^* mice (Mori–Akiyama et al., 2003). *Fgf9* has been reported to be regulated by *Sox9* in relation to sex determination (Loke et al., 2014). Thus, this tight link between *Sox9* and *Fgf9* may occur during palatogenesis.

*Fgf9* cooperates closely with other FGF family members during palatogenesis. *Fgf9-Fgfr2* regulate cell proliferation (Chang et al., 2018). Moreover, a complete loss of epithelial *Fgfr2* leads to small palates and delayed elevation resembling that in *Fgf9^–/–^* mice (Rice et al., 2004). Furthermore, *Fgf9* and *Fgf18* have overlapping functions in skeletal development and they have been shown to interact in families with cases of cleft lip and palate (Wang et al., 2013; Hung et al., 2016). The gene–gene interaction test showed mesenchyme-specific *Fgf18* knockout mice with a shortened mandible and cleft palate caused by impaired palatal elevation (Liu et al., 2002; Yue et al., 2020).

Conditional knockout of *Has2*, the gene encoding the hyaluronic acid synthase 2, results in small shelf size and failed palatal elevation, resembling the conditions in *Fgf9^–/–^* mice (Yonemitsu et al., 2020). Moreover, HA is a downstream target of *Fgfs* (Nikitovic et al., 2013; Bohrer et al., 2014; McCarthy et al., 2016; Virakul et al., 2016; Wolk et al., 2020). Many studies have shown that FGF family members share an intimate regulatory network with *Has2*, and *Fgf9* may be a critical factor connecting the FGF family and *Has2*.

Therefore, the *Fgf9^–/–^* embryos obtained from pregnant *Fgf9^+/–^* mice that had mated with male *Fgf9^+/–^* mice, which had been established using *Fgf9*^*F/+*^ (F indicates a floxed allele) and *Ddx4-Cre* lines, provide an excellent model for studying these pathways and the genetic crosstalk. The *Ddx4-Cre* (also known as *Vasa-Cre*) mouse model, which is currently rarely utilized in research on palatogenesis, is promising for mediating global deletion. The *Fgf9^+/–^* mice generated from *Ddx4-Cre* recombination harbored “flox” deletion in their germ cells. This contributes to its efficiency and reliability regarding globally deleting a specific gene in the offspring comparing with using *EIIa-Cre* and *Nestin-Cre* (Gallardo et al., 2007). The major drawback is the lower fertility, especially in female *Fgf9^+/–^* mice, which makes obtaining adequate sample sizes more challenging. However, its efficiency and reliability make it an optimal choice for starting to explore a specific gene’s function in craniofacial development. Furthermore, global deletion lays a solid foundation for further investigation based on conditional knockouts.

The discussion below primarily focuses on how *Fgf9* mediates palatogenesis based on our current findings and the findings of related studies.

### *Fgf9* Plays an Essential Role in the Epithelial–Mesenchymal Communication (EMC) That Underlies Palatal Growth and Elevation

As early as E12.5, *Fgf9* is already found in the epithelium of palatal shelves. During vertical palatal growth, *Fgf9^–/–^* shelves failed to form regular morphology. Meanwhile, the mesenchymal cell density increased and the palatal elevation was delayed. After elevation, the bilateral malformed shelves failed to meet each other in the midline of the oral–nasal cavity. These successive abnormalities result from impaired cell proliferation and HA accumulation in the mesenchyme, which reveals that EMC is key during palatogenesis. This communication is based on the overlaying of the *Fgf9* and *Fgfr2* expression patterns in the epithelium of the developing palate and the resultant in modulation of palatal cell proliferation by *Fgf9*–*Fgfr2* (Huang et al., 2016; Weng et al., 2018). Additionally, *Fgfr2* combined with *Fgf10*, which is expressed in the palatal mesenchyme, along with *Shh*, plays an essential role in EMC (Colvin et al., 2001; Li et al., 2019). Studies have shown that *Fgf9* participates in the *Shh*–*Fgfr2*–*Fgf10* pathway during lung, cecal, and inner ear development (Colvin et al., 2001; Pirvola et al., 2004; Al Alam et al., 2012). Thus, future research should investigate the participation of *Fgf9* in EMC underlying palatogenesis.

### *Fgf9*-Induced Cell Proliferation and Hyaluronic Acid Accumulation Are of Comparable Importance in Palatal Vertical/Horizontal Growth and Elevation

For decades, there was a consensus that cell proliferation is the critical factor that induces palatal growth and that it is a requisite for palatal elevation (Li et al., 2019). However, in recent years continuous research has indicated the possible role of HA accumulation in ECM expansion; HA accumulation is no longer merely an internal force for palatal elevation but has emerged as a potential promoter of tissue expansion during palatal growth (Chiquet et al., 2016; Li et al., 2017; Lan et al., 2019; Yonemitsu et al., 2020). Combining recent related research with our results, we propose that impaired cell proliferation is not the only reason for aberrant palatal growth. If smaller palatal shelves occur as a result of reduced cell proliferation, the cell density in *Fgf9^–/–^* would be similar to that of the *Wildtype*, which was not the case. This conclusion is in line with the previous finding in lung development that *Fgf9* regulates embryonic organ size by regulating mesenchymal expansion (Colvin et al., 2001). Moreover, the decrease in HA accumulation corresponds to the increase in cell density from E12.5 to E13.5. The evidence collectively indicates the unique and irreplaceable role of HA accumulation in palatal growth.

The aberrant palatal growth impairs subsequent palatal elevation in the *Fgf9^–/–^* embryos. At E14.5 in *Fgf9^–/–^* embryos, mesenchymal cell proliferation rates in the middle and posterior regions remained low, which impaired palatal remodeling. Further, the *Fgf9^–/–^* shelves that exhibited timely elevation had impaired cell proliferation compared to the *Fgf9^+/–^* shelves. This indicates the impairment after *Fgf9^–/–^* palatal elevation. Additionally, apoptosis was not observed in the distal portion of the palatal shelves which verified that palatal shelf remodeling does not involve apoptosis but a retraction or migration of cells instead.

Regarding HA accumulation during palatal elevation, it was decreased not only in the *Fgf9^–/–^* but also in the *Fgf9^+/–^* mice at E13.5. Intriguingly, there were varying degrees of delayed palatal in *Fgf9^+/–^* embryos, with some of the embryos showing similar palatal elevation to *Fgf9^–/–^* embryos, but without exhibiting the tongue-related obstacles to palatal elevation. Thus, it is beyond all doubt that decreased HA accumulation owing to partial ablation of *Fgf9* can lead to delayed elevation. However, it is certainly not the sole reason for delayed elevation in the *Fgf9^–/–^* mice given the observation of tongue-related obstruction and, eventually, failed contact of the palatal shelves. *In vitro* organ culture without the tongue and mandible is required to confirm this potential conclusion.

### *Fgf9* Affects the Oral Volume Available for Palatal Elevation by Influencing Tongue Size and Descent

To further explore the factors involved in palatal elevation, the histomorphology of *Wildtype*, *Fgf9^+/–^*, and *Fgf9^–/–^* embryos was analyzed. The decrease in oral volume was a major cause of the disordered palatal elevation in *Fgf9^–/–^* model. Compared to the *Wildtype* mice, the tongue size and height in the *Fgf9^+/–^* mice decreased, making way for palatal shelf movement. However, a similar phenomenon did not appear in the *Fgf9^–/–^* mice; in fact, even a larger tongue size and height were observed compared to the *Wildtype mice.* The *Fgf9^–/–^* tongue might result from either abnormal tongue movement or intrinsic tongue malformation.

The *Fgf9^+/–^* mice underwent delayed palatal elevation. The shelves elevated in a wave-like manner from the posterior to the anterior palate, which is in line with the opinion of Professor Bush and Professor Jiang regarding *Wildtype* mice (Bush and Jiang, 2012). However, *Fgf9^–/–^* shelves asynchronously elevate from the anterior and middle of the palate with the posterior part unelevated, unlike in *Fgf9^+/–^* mice. The divergence might arise from abnormal tongue descent or tissue remodeling in the posterior palatal shelves.

### *Fgf9* Promotes Mandibular Enlargement by Enhancing Cell Proliferation in Meckel’s Cartilage and Intramembranous Ossification Site in Order to Provide Adequate Oral Volume

Besides the tongue descent that allows more space for palatal elevation, the mandible goes through a twisting motion to create space for unelevated shelves to find their way to the horizontal position (Yu and Ornitz, 2011). In *Fgf9^–/–^* mice, the smaller mandibular height decreases the oral volume and, along with the twisting motion, decreases the space for the anterior shelves to “flip-up” and for the mid-posterior shelves to undergo remodeling. *Fgf9* is non-uniformly distributed and performs diverse functions in various developmental regions and at the various stages of endochondral and intramembranous ossification (Colvin et al., 1999; Govindarajan and Overbeek, 2006; Su et al., 2014). *Fgf9* is located in the perichondrium, periosteum, trabecular bone, and related mesenchyme of the mandible, and it exhibits an identical expression pattern to that in developing limb cartilage (Hung et al., 2007). Cell proliferation is impaired in the perichondrium of *Fgf9^–/–^* Meckel’s cartilage. This might lead to aberrant cartilage growth potential (Yokohama–Tamaki et al., 2011), resulting in a smaller mandibular size. It is known that Meckel’s cartilage and condylar cartilage make different but equal contributions to mandibular development (Ramaesh and Bard, 2003; Yokohama–Tamaki et al., 2011). Future research is encouraged to focus on the mechanism of aberrant mandibular development in *Fgf9^–/–^* mice and whether condylar cartilage growth plays a role in palatal elevation.

In addition to the Meckel’s cartilage and condylar cartilage development, intramembranous ossification also contributes to the growth of the mandible (Parada and Chai, 2015). *Fgf9* enhanced the mesenchymal cell proliferation rate at the initial site of intramembranous ossification. Moreover, *Fgf9* even participated in the strengthening of the mandibular bone by enhancing the proliferation of the embedded cells. The separated and thin trabecular bone observed in the histological analysis of *Fgf9^–/–^* embryos provide further support for this. Nevertheless, further extensive research should be conducted on the functions of *Fgf9* during mandibular formation.

## Conclusion

In this study, we generated *Fgf9* knockout mice with a cleft secondary palate and a small mandible. We dissected the mouse embryos and investigated the possible functions of *Fgf9* during palatal development. We concluded that *Fgf9* regulates cell proliferation, which involves the epithelial–mesenchymal communication. This facilitates vertical and horizontal palatal growth before elevation and contact, respectively. Additionally, by ensuring hyaluronic acid accumulation in the extracellular matrix space, *Fgf9* is involved in palatal shelf expansion and the timely “flipped up” of the anterior shelves and the remodeling of the mid-posterior shelves. Moreover, *Fgf9* benefits mandibular growth, which provides the elevating shelves with adequate space, by promoting Meckel’s cartilage growth and intramembranous ossification. Finally, *Fgf9* regulates tongue size and descent, which allows more space for palatal elevation.

## Data Availability Statement

The original contributions presented in the study are included in the article/supplementary material, further inquiries can be directed to the corresponding author/s.

## Ethics Statement

The animal study was reviewed and approved by the Animals Committee of Shanghai Ninth People’s Hospital.

## Author Contributions

RL made substantial contribution to generation of the mouse model, data collection, analysis and interpretation, and drafting the manuscript. YS generated the mouse model. ZC made substantial contribution to the preparation of the experimental images. MZ made substantial contribution to the literature review. YS and XY made substantial contribution to the data collection and analysis. ZC and MW made substantial contribution to the concept and design of the study, data analysis, and critical revision for the important intellectual content. All authors read and approved the final manuscript.

## Conflict of Interest

The authors declare that the research was conducted in the absence of any commercial or financial relationships that could be construed as a potential conflict of interest.
